# Establishment of a FDC-P1 Murine Cell Line with Human KIT N822K Gene Overexpression

**DOI:** 10.32607/actanaturae.10938

**Published:** 2020

**Authors:** E. R. Vagapova, T. D. Lebedev, V. I. Popenko, O. G. Leonova, P. V. Spirin, V. S. Prassolov

**Affiliations:** Engelhardt Institute of Molecular Biology, Russian Academy of Sciences, Moscow, 119991 Russia

**Keywords:** receptor tyrosine kinase KIT, FDC-P1, acute myeloid leukemia (AML), KIT N822K, stromal cells

## Abstract

The mechanism of resistance of leukemia cells to chemotherapeutic drugs remains poorly understood. New model systems for studying the processes of malignant transformation of hematopoietic cells are needed. Based on cytokine-dependent murine acute myeloid leukemia (AML) FDC-P1 cells, we generated a new cell line with ectopic expression of the *KIT *gene encoding mutant human receptor tyrosine kinase (N822K). We investigated the role played by overexpression of the mutant *KIT *in the survival of leukemia cells and their sensitivity to therapeutic drugs. We also generated a co-culture system consisting of FDC-P1 murine leukemia cells and a HS-5 human stromal cell line. Our data can be used for a further comprehensive analysis of the role of* KIT *N822K mutation in the cellular response to anti-leukemic drugs, growth factors, and cytokines. These data are of interest in the development of new effective therapeutic approaches to the treatment of acute leukemia.

## INTRODUCTION

Expression of the *KIT *gene encoding receptor tyrosine kinase is often observed in various malignant diseases: leukemia, neuroblastoma, gastrointestinal stromal tumors (GISTs), mast cell tumors, and melanoma [1-3]. In normal hematopoiesis, *KIT *is expressed in a subpopulation of immature hematopoietic progenitor cells positive for CD34 (a marker of stem cells and early progenitor cells) [4].

Mutations in the KIT kinase gene are found in cells from patients with acute myeloid leukemia (AML) and mastocytosis; most frequently those are D816V and N822K mutations localized in the tyrosine kinase domain and leading to constitutive activation of tyrosine kinase. The activating D816V and D814V mutations significantly reduce cell sensitivity to the KIT inhibitor imatinib (Gleevec; Novartis Pharma AG, Basel, Switzerland) both *in vitro *and *in vivo *[5, 6]*. *Mutations in the KIT kinase domain are associated with an increased relapse rate of FAB M2 AML after chemotherapy [7]. Imatinib is a highly selective inhibitor of the Abl, BCRABL, PDGFRα/β, and KIT kinases; it is used in BCRABL- positive leukemia and GISTs carrying mutations in *KIT *and *PDGFR*. Imatinib is used in combination with other anti-leukemic drugs in AML. Imatinib was shown to have a strong effect on AML cells in high-risk populations. However, high-dose imatinib monotherapy often leads to severe cytopenia, and, therefore, is not recommended [8].

A new, continuous cell line overexpressing mutant human kinase *KIT *(*N822K) *has been generated using FDC-P1 cytokine-dependent murine acute myeloid leukemia cells with a low expression level of the wildtype* KIT *gene. We investigated the role of the mutation, which is located in the tyrosine kinase domain, in maintaining the malignant status of leukemic cells: their survival, sensitivity to drugs, and growth rates when in contact with stromal cells.

## EXPERIMENTAL

**Cell cultures**

Continuous FDC-P1 cells were cultured in IMDM supplemented with 10% fetal bovine serum (FBS), 100 U/ml penicillin, 100 μg/ml streptomycin, 1 mM sodium pyruvate, 2 mM *L*-glutamine, and a 7% WEHI-3B conditioned medium containing mouse interleukin-3 (IL-3) at 37°C and 5% CO_2_. HS-5 human stromal cells   were cultured in a RPMI-1640 medium containing 10% FBS, 100 U/ml penicillin, 100 μg/ml streptomycin, 1 mM sodium pyruvate, and 2 mM *L*-glutamine. All reagents were purchased from Gibco, Thermo Fisher Scientific (USA). Cell lines were obtained from the Heinrich Pette Institute, Leibniz Institute for Experimental Virology (HPI, Hamburg, Germany), and tested for the absence of mycoplasma contamination.

**Immunocytochemical analysis**

For qualitative analysis, FDC-P1 cells were fixed with 4% paraformaldehyde in 0.1 M phosphate buffered saline (PBS) for 15 min then washed with PBS and treated with PBS containing 0.2% Triton X-100 and human KIT-specific antibodies conjugated to PerCP/Cy5.5 (PerCP/Cy5.5, Abcam, ab157320, 1 : 50). After washing with PBS, the cells were embedded in a SlowFade Gold mounting medium (Invitrogen, USA, s36936) containing 1 μg/ml DAPI (Sigma-Aldrich, USA); the slides were sealed with nail polish. The samples were analyzed using a Leica TCS SP5 confocal microscope (Leica, Germany) and an HCX PLAPO CS lens. For quantitative assessment of the staining intensity, the FDC-P1 cells were treated with human KIT-specific antibodies conjugated to PerCP/Cy5.5 (PerCP/Cy5.5, Abcam, ab157320, 1 : 50) and analyzed on an LSRFortessa flow cytometer (BD Biosciences). Data analysis was performed using the FlowJo software.

**Quantitative real-time PCR and primer design**

RNA was isolated using the Trizol reagent (Invitrogen, USA) according to the manufacturer’s protocol. RNA concentration and purity were determined on a spectrophotometer (NanoDrop). Complementary DNA was synthesized using a reverse transcription kit (Thermo Fisher Scientific, USA) (random primers). Real-time PCR was performed using Maxima SYBR Green Supermix (Thermo Fisher Scientific) on a CFX96 Real-Time System (Bio-Rad, USA). Expression of the target genes was normalized to that of *b-actin *in each sample. The Ct and relative expression level were calculated using the Bio-Rad CFX manager 3.1 software. At least three replicates were used in each experiment. Primers were designed in the Primer-Blast (NCBI, USA) using the following parameters: amplicon length, 50 to 200 bp; primer annealing temperature, 57°C. The energy characteristics of the primer pairs were checked using the OligoAnalyzer tool (idtdna) to exclude the formation of high-energy hairpin structures and dimers (more than 10 kJ). The primer sequences were as follows: beta-actin forward 5’-TCAAGATCATTGCTCCTCCTGA- 3’; beta-actin reverse 5’-ACGCAGCTCAGTAACAGTCC-3’; musKIT forward 5’-CCATAGACTCCAGCGTCTTCC-3’; musKIT reverse GCCTGGATTTGCTCTTTGTTGTT-3’; hum- KIT forward 5’-CCACCCTGGTCATTACAGAA-3’; humKIT reverse 5’-CTCCAGGTTTCATGTCCATG-3’.

**Statistical analysis**

All tests and the deviation calculation were performed using the GraphPad prism software.

## RESULTS AND DISCUSSION

**Generation of continuous murine leukemia cells overexpressing a mutant human** *KIT N822K* **gene**

**Fig. 1:**
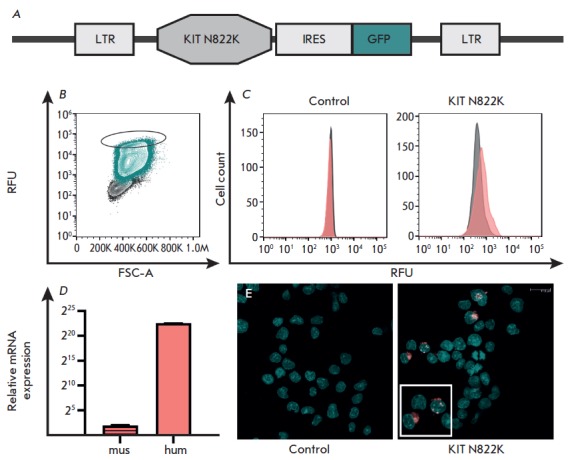
Schematic representation of the vector for the expression of the human *KIT N822K *gene (*A*); isolation of the population of cells with high fluorescence intensity in the FITC channel (highlighted with an ellipse) by cell sorting (*B*), the population of non-transduced FDC-P1 cells is shown in gray, cells transduced with the vector carrying *KIT N822K* are shown in green; surface KIT receptor expression in the control cells and cells showing overexpression of the mutant human *KITN 822K *(*C*); cells were treated with PerCP5.5-conjugated monoclonal antibodies to human KIT and analyzed by flow cytometry: untreated cells are shown in gray, cells treated with human KIT-specific antibodies are shown in pink; the expression level of the murine *KIT *(mus) and human *KIT *(hum) genes in FDC-P1 cells with overexpression of *KIT N822K *according to the qPCR data (*D*); immunocytochemical evaluation of the expression of the human KIT receptor gene (pink) in FDC-P1 cells on a confocal microscope (*E*); nuclei are stained with DAPI (green) (*E*)

In order to analyze the oncogenic potential of KIT receptor tyrosine kinase with a N822K mutation in the kinase domain (and its ability to influence the proliferation of leukemia cells of myeloid origin in particular), the FDC-P1 N822K cell line was obtained. For this purpose, IL-3-dependent mouse FDC-P1 cells were transduced with a retroviral vector expressing human KIT receptor tyrosine kinase with the N822K mutation. The vector also contianed the GFP reporter gene (*Fig. 1A*). The vector was kindly provided by Mrs. Carol Stocking (HPI, Hamburg, Germany). The control cell line was transduced with the original retroviral vector lacking* KIT N822K*.

The population of cells with the highest fluorescence intensity in the FITC channel, which corresponds to the high *GFP *expression level, was isolated by cell sorting (S3e Cell Sorter, Bio-Rad) (*Fig. 1B*). The expression level of *KIT *was determined by real-time PCR in the selected cells (*Fig. 1D*). FDC-P1 N822K cells express the KIT protein on their surface (*Fig. 1C*). The presence of the human KIT protein in FDC-P1 KIT N822K cells was confirmed by confocal microscopy, while no human KIT expression was detected in the FDC-P1 cells transduced with the control vector: neither at the mRNA nor the protein level (*Fig. 1 C, E*).

***KIT N822K *mutation results in IL-3- independent growth of FDC-P1 cells**

**Fig. 2:**
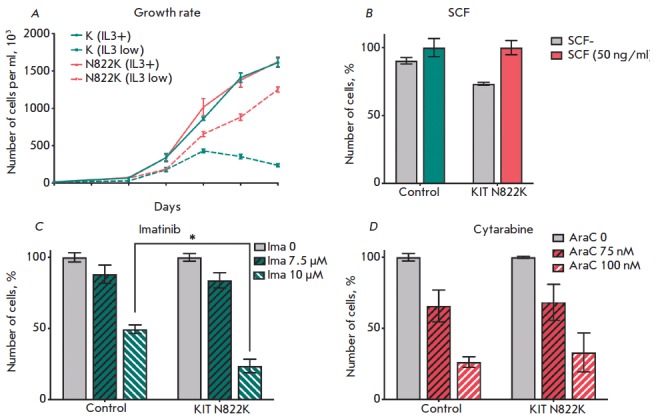
Growth curve of FDC-P1 cells in the presence of IL-3 (solid line) and in a reduced IL-3 content (dotted line) (*A*); the number of FDC-P1 cells treated with recombinant SCF for 4 days per milliliter (*B*); the number of FDC-P1 cells as a percentage relative to the untreated cells on day 3 after addition of imatinib (*C*) and cytarabine (*D*) drugs. *p < 0.05

The control cells and FDC-P1 N822K cells were seeded at the same density. Cell counting was performed for 6 days. Introduction of the mutant *KIT N822K *did not affect the growth rate of FDC-P1 cells in the presence of IL-3 (*Fig. 2A*).

Overexpression of the mutant *KIT *in FDC-P1 cells leads to their factor-independent growth (*Fig. 2A*). The growth rate of FDC-P1 control cells is significantly lower in a medium with a reduced content of a IL- 3-conditioned medium (0.25%).

We observed no significant changes in the cell growth rate after treatment with the KIT–SCF ligand (Abcam) (*Fig. 2B*). The control and FDC-P1 N822K cells were treated with the antitumor drugs imatinib (5 and 10 μM) and cytarabine (75 and 100 nM). The amount of cells was counted on day 3 after addition of the drugs. The imatinib concentration inhibiting the growth of the FDC-P1 control cells by 50% (IC_50_) was 10 μM. FDC-P1 cells overexpressing *KIT N822K *turned out to be more sensitive to this drug concentration (*Fig. 2C*). Cell sensitivity to cytarabine remained the same as that in the control cells (*Fig. 2D*).

Our data are in line with the finding that mutation in the KIT tyrosine kinase domain, in particular D816V, enhances cell sensitivity to imatinib [9].

**Generation of a co-culture of leukemic and stromal cells**

The factors that facilitate the production of stromal cells are involved in the stimulation of hematopoietic cell proliferation, the regulation of the cell cycle, and apoptosis. Meanwhile, the processes occurring when stromal cells come into contact with leukemic cells remain poorly understood.

**Fig. 3:**
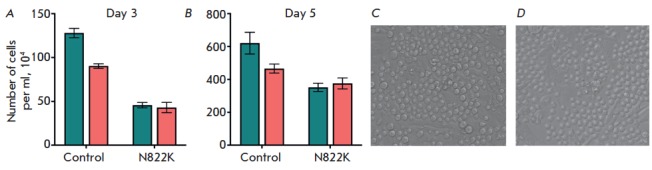
The number of FDC-P1 cells cultured in the absence (green) and presence of HS-5 human stromal cells (pink) for 3 (*A*) and 5 (*B*) days; images of FDC-P1 cells co-cultured with HS-5 cells: control (*C*) and N822K (*D*) cells. Longitudinal stromal cells adhere to the plate bottom, while round FDC-P1 cells remain in suspension

Continuous HS-5 human stromal cells were seeded at 5,000 cells per well. On the next day, the culture medium of HS-5 cells was changed to IMDM containing FDC-P1 cells (500 cells per well). The number of cells in the suspension fraction was counted 3 and 5 days after seeding. Direct interaction between leukemic and stromal cells leads to a reduction in the growth rate of the control FDC-P1 cells (*Fig. 3 A, B*) but not the FDC-P1 cells overexpressing *KIT N822K*.

Cytokines and growth factors produced by stromal cells (including HS-5 cells) can modulate *KIT *expression in co-cultured leukemic cells [10]. Apparently, the growth rate of FDC-P1 cells with ectopic expression of *KIT N822K *does not change upon overexpression of the kinase. Moreover, the growth rate can vary due to differences in the adhesion of control FDC-P1 cells and FDC-P1 N822K cells.

## CONCLUSION

The IL3-dependent murine cell line FDC-P1 is widely used to study the oncogenic effect of kinases, transcription factors, as well as the effectiveness of anti-leukemic drugs [11, 12]. We have obtained and characterized the FDC-P1 cell line overexpressing the mutant human *KIT N822K *gene. It has been shown that N822K mutation in *KIT *increases the sensitivity of FDC-P1 cells to imatinib. The D419A mutation in the extracellular domain of the KIT receptor also increases cell sensitivity to imatinib [9]. It was shown that the growth rate of control cells that come into contact with the stroma decreases, which is not typical of FDC-P1 cells expressing the mutant *KIT N822K *gene. Closer attention should be paid to the study of the mechanisms of interaction between leukemic and stromal cells in order to establish any possible contribution of stromal cells to the response of leukemic cells to chemotherapeutic agents. Our model can be used to test various anti- leukemic drugs, including co-cultivation of leukemic and stromal cells.

## Abbreviations

SCF: stem cell factor
AML: acute myeloid leukemia
FAB M2: acute myeloblastic leukemia with maturation (AML subtype according to the French–American–British classification)
CD34: cluster of differentiation 34
HPI: Heinrich Pette Institute, Leibniz Institute for Experimental Virology
GFP: green fluorescent protein.
